# Mesenchymal stromal cells reset the scatter factor system and cytokine network in experimental kidney transplantation

**DOI:** 10.1186/s12865-014-0044-1

**Published:** 2014-10-03

**Authors:** Marilena Gregorini, Francesca Bosio, Chiara Rocca, Valeria Corradetti, Teresa Valsania, Eleonora Francesca Pattonieri, Pasquale Esposito, Giulia Bedino, Chiara Collesi, Carmelo Libetta, Francesco Frassoni, Antonio Dal Canton, Teresa Rampino

**Affiliations:** Unit of Nephrology, Dialysis and Transplantation, Fondazione, IRCCS Policlinico San Matteo and University of Pavia, viale Golgi 19, 27100 Pavia, Italy; Unit of Nephrology, Dialysis and Transplantation, Fondazione, IRCCS Policlinico San Matteo, viale Golgi 19, 27100 Pavia, Italy; ICGEB, International Centre for Genetic Engineering and Biotechnology, Trieste, Italy; Stem Cells Therapy and Hemato-Oncology, S.Martino Hospital, 16100 Genoa, Italy

**Keywords:** Mesenchymal stromal cells, Acute kidney rejection, Experimental model, Hepatocyte growth factor, Macrophage stimulating protein, Scatter factors

## Abstract

**Background:**

In former studies we showed in a rat model of renal transplantation that Mesenchymal Stromal Cells (MSC) prevent acute rejection in an independent way of their endowing in the graft. In this study we investigated whether MSC operate by resetting cytokine network and Scatter Factor systems, i.e. Hepatocyte Growth Factor (HGF), Macrophage Stimulating Protein (MSP) and their receptors Met and RON, respectively.

**Methods:**

MSC were injected into the renal artery soon after reperfusion. Controls were grafted untreated and normal rats. Rats were sacrificed 7 days after grafting. Serum and renal tissue levels of IFN-γ, IL-1, IL-2, IL-4, IL-6, IL-10, MSP/RON, HGF/Met systems, Treg lymphocytes were investigated.

**Results:**

In grafted untreated rats IFN-γ increased in serum and renal tissue and IL-6 rose in serum. MSC prevented both the phenomena, increased IL-10 serum levels and Treg number in the graft. Furthermore MSC increased serum and tissue HGF levels, Met tubular expression and prevented the suppression of tubular MSP/RON expression.

**Conclusions:**

Our results demonstrate that MSC modify cytokine network to a tolerogenic setting, they suppress Th1 cells, inactivate monocytes/macrophage, recruit Tregs. In addition, MSC sustain the expression of the Scatter Factor systems expression, i.e. systems that are committed to defend survival and stimulate regeneration of tubular cells.

## Background

Mesenchymal Stromal Cells (MSC) are pluripotent cells that differentiate into various mature cell types [1,2]. A distinctive property of MSC is that they are not immunogenic [3,4], and inhibit cell and antibody-mediated immunity in several ways, including the induction of T regulatory cell differentiation [5-14]. In a rat model of kidney transplantation we found that MSC injected in the graft improved its function and attenuated renal injury, reducing significantly tubulitis, vasculitis, glomerulitis and immune cell infiltration. Furthermore, we traced MSC in the recipient tissues and found an irrelevant number of them in the kidney. The last finding suggests that mediators account for the protection provided by MSC to the renal graft [15]. A renal protective effect of MSC was shown also in a mouse model of renal transplantation, in which MSC suppressed rejection when they were infused before transplantation, while post-transplant infusion worsened graft outcome [16]. Interestingly, MSC injected before transplantation were found in lymphoid organs and did not localize in the graft, confirming the need for some mediation of MSC effects. Indeed a mediated action of MSC has been proved in diverse experimental disease models [17-23], and cytokines have been proposed to play the role of effectors [24-32]. However, as yet no evidence has been given that MSC modify the cytokine network in the setting of renal graft, so that we have felt it interesting to test the hypothesis in the present study.

In addition, we have thought of the Scatter Factors as a system that could be reset by MSC and provide protection in the renal graft model. The Scatter Factor (SF) system consists of Hepatocyte Growth Factor (HGF) and Macrophage Stimulating Protein (MSP) and their receptors Met and RON respectively. There are several reasons to think that MSC operate through the SF system. In fact, the SFs are expressed in normal kidney [33-35] and participate in the regulation of cell growth and inflammation in various renal diseases including autoimmune forms, e.g. they stimulate renal cell growth and modulate monocyte traffic in the kidney [36-42]. Furthermore, we demonstrated that in experimental anti Thy-1 nephritis MSC improve renal injury by modulating SFs [43].

In summary, we investigated whether MSC injected into the renal graft modify the cytokine network and SFs in a way that fits with the concept that these systems are the effectors of MSC-induced graft protection.

## Results and discussion

### Characterization of rat MSC

As detailed elsewhere the MSC used in the present study were isolated from Sprague Dawley EGFP rats and differentiated into osteogenic and adipogenic cells. Flow fluorocytometry analysis showed that MSC were positive for CD90 (≥95%) and CD73 (≥95%) and were negative for CD45 (<5%), CD11b (<5%), CD34 (<5%), CD79 (<5%).

### Tissue and serum levels of cytokines

The expression in renal tissue of IFN-γ, IL-10, IL-6, IL-2, IL-1 was significantly increased in grafted kidneys not injected with MSC compared with native kidneys. MSC injection attenuated significantly the rise of IFN-γ and caused a further increase of IL-10. MSC did not modify tissue levels of any else cytokine. IL-4 tissue levels were similar in all groups (Table 1). In MSC untreated rats grafting was associated with a rise in serum levels of all tested cytokines, except for IL-4. MSC treatment reduced serum IFN-γ and IL-6 and increased IL-10 compared to untreated rats (Table 2).

**Table 1:** **Cytokine levels in renal tissue**

**pg/mg**	**A**	**B**	**C**	**B vs C**	**p**
**INF-γ**	**35.2 ± 0.02** ^**§**^	**206.3 ± 64.4°**	**116.7 ± 56.1**	**↓**	^**§**^ **p < 0.005 vs B, C; °p < 0.005 vs C**
**IL-10**	**8.6 ± 1.4** ^**§**^	**25.53 ± 0.7°**	**42.2 ± 10.6**	**↑**	^**§**^ **p < 0.005 vs B, C; °p < 0.001 vs C**
**IL-6**	**117.7 ± 11.36°**	**245.5 ± 28.8**	**283.0 ± 32.7**	**NS**	**°p < 0.001 vs B, C**
**IL-2**	**140.6 ± 0.14°**	**227 ± 93.1**	**262 ± 103.4**	**NS**	**°p < 0.005 vs B, C**
**IL-1**	**235.8 ± 134.4°**	**505.4 ± 219.7**	**493 ± 142**	**NS**	**°p < 0.005 vs B, C**
**IL-4**	**13.05 ± 1.4**	**26.6 ± 6**	**32.8 ± 5.2**	**NS**	**NS**

Groups are defined in Figure 8. A indicates cytokine levels in kidneys of healthy rats, B and C indicate cytokine levels in renal tissue of recipient rats 7 days after transplantation. Data represent means ± SD. The symbols § and ° indicate the different statistical power.

**Table 2:** **Serum cytokine levels**

**pg/ml**	**A**	**B**	**C**	**B vs C**	**p**
**INF-γ**	**14 ± 0.08°**	**151.2 ± 78.6** ^**§**^	**86.7 ± 32.1**	**↓**	**°p < 0.0001 vs B, C; ** ^**§**^ **p < 0.05 vs C**
**IL-10**	**0°**	83.9 ± 9.7*	**139.1 ± 20.4**	**↑**	°p < 0.0001 vs B, C; *p < 0.05 vs C
**IL-6**	**0°**	**183 ± 61.01** ^**#**^	**14.09 ± 0.06**	**↓**	**°p < 0.0001 vs B, C; ** ^**#**^ **p < 0.05 vs C**
**IL-2**	**0°**	**61 ± 24.7**	**94.6 ± 61**	**NS**	**°p < 0.05 vs B, C**
**IL-1**	**0°**	**1515 ± 613**	**1843 ± 670**	**NS**	**°p < 0.005 vs B, C;**
**IL-4**	**0**	**0**	**0**	**NS**	

Groups are defined in Figure 8. A indicates serum cytokine levels in healthy rats. B and C indicate serum cytokine levels in recipient rats 7 days after transplantation. Data are means ± SD. The symbols §, °, *, # indicate the different statistical power.

### Tubular necrosis and tubular cell proliferation

**Figure 1:**
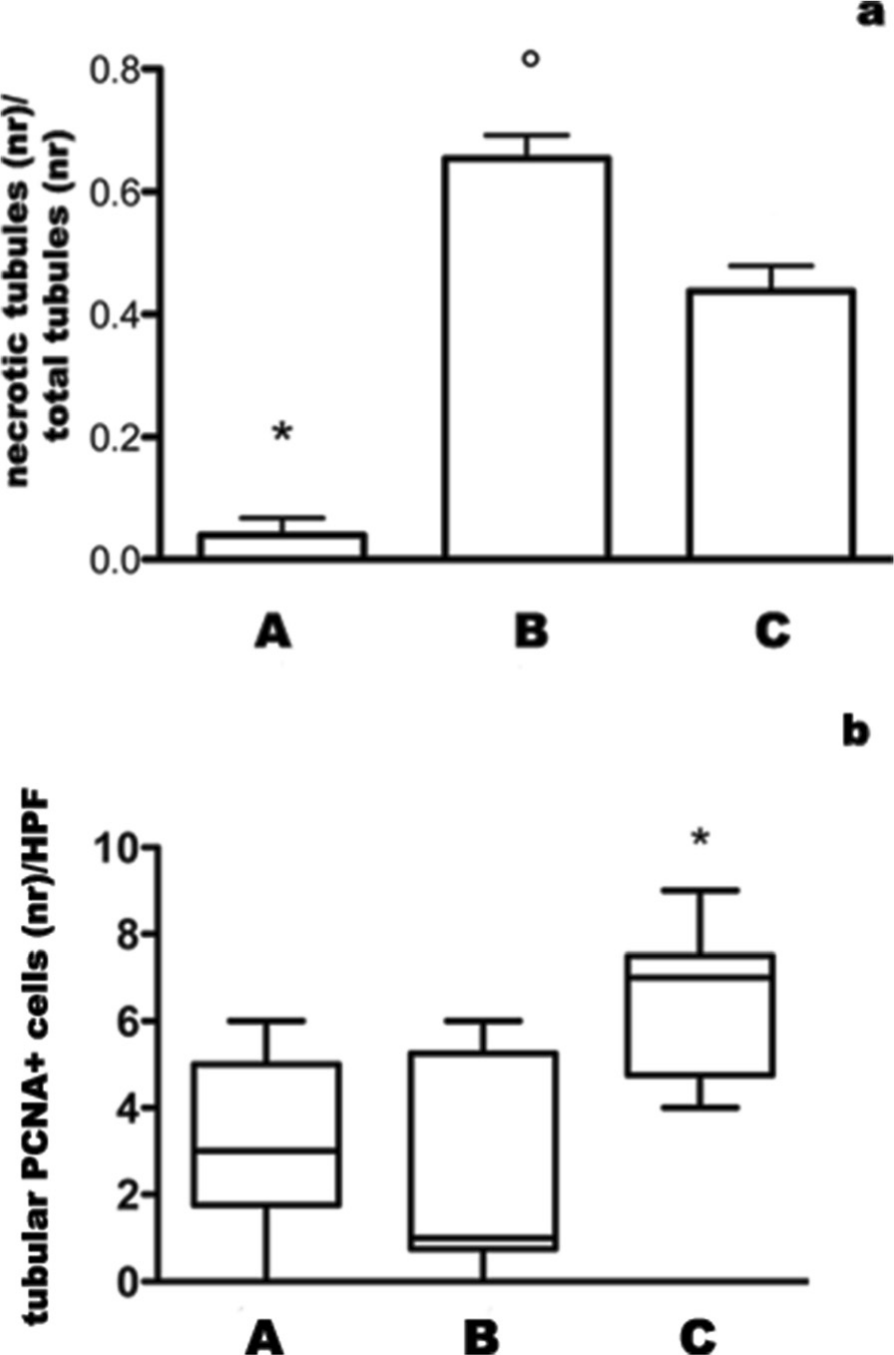
**Tubular necrosis and tubular cell proliferation.** Groups are defined as in Figure 8. Panel **a**: Columns represent the ratio between necrotic tubules number and total counted tubules (bars are SD). °p < 0.001 vs C; *p < 0.0001 vs B and C. Panel** b**: Boxes represent 25-75% percentile of tubular PCNA-positive cell number/HPF (bars are medians, whiskers: min to max). *p < 0.005 vs A and B.

Tubules with necrotic cells were significantly less in grafts injected with MSC compared to untreated grafts (Figure 1, panel a). The expression of PCNA was suppressed in grafts not injected with MSC, but it was spared by MSC treatment (Figure 1, panel b).

### Foxp3 cells infiltrate

**Figure 2:**
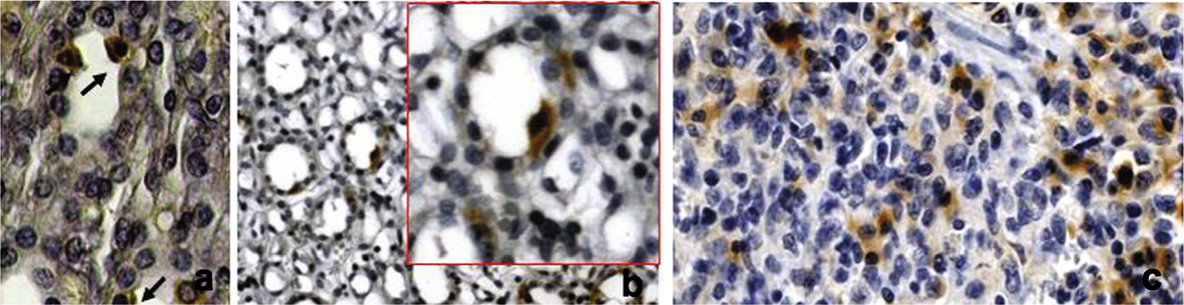
**Foxp3 positive cells.** Foxp3 staining of representative kidney and spleen sections. Panel** a** shows Foxp3 positive cells in cortical tubules after 7 days from transplantation in rats of group C (Magnification X400). Panel** b** shows Foxp3 positive cells in medullary tubules after 7 days from transplantation in rats of group C (Magnification X 200 and X400). Panel** c** shows Foxp3 positive cells in spleen after 7 days from transplantation in rats of group C (Magnification X 200).

In the grafted kidneys not injected with MSC we found few Foxp3 positive cells (2 ± 1/section) and the number increased in MSC treated rats (10 ± 5/section, p < 0.05). In the spleen we detected 15 ± 10 cells/section (Figure 2).

### HGF/Met System

#### Serum and renal tissue HGF levels, HGF mRNA expression in kidneys

**Figure 3:**
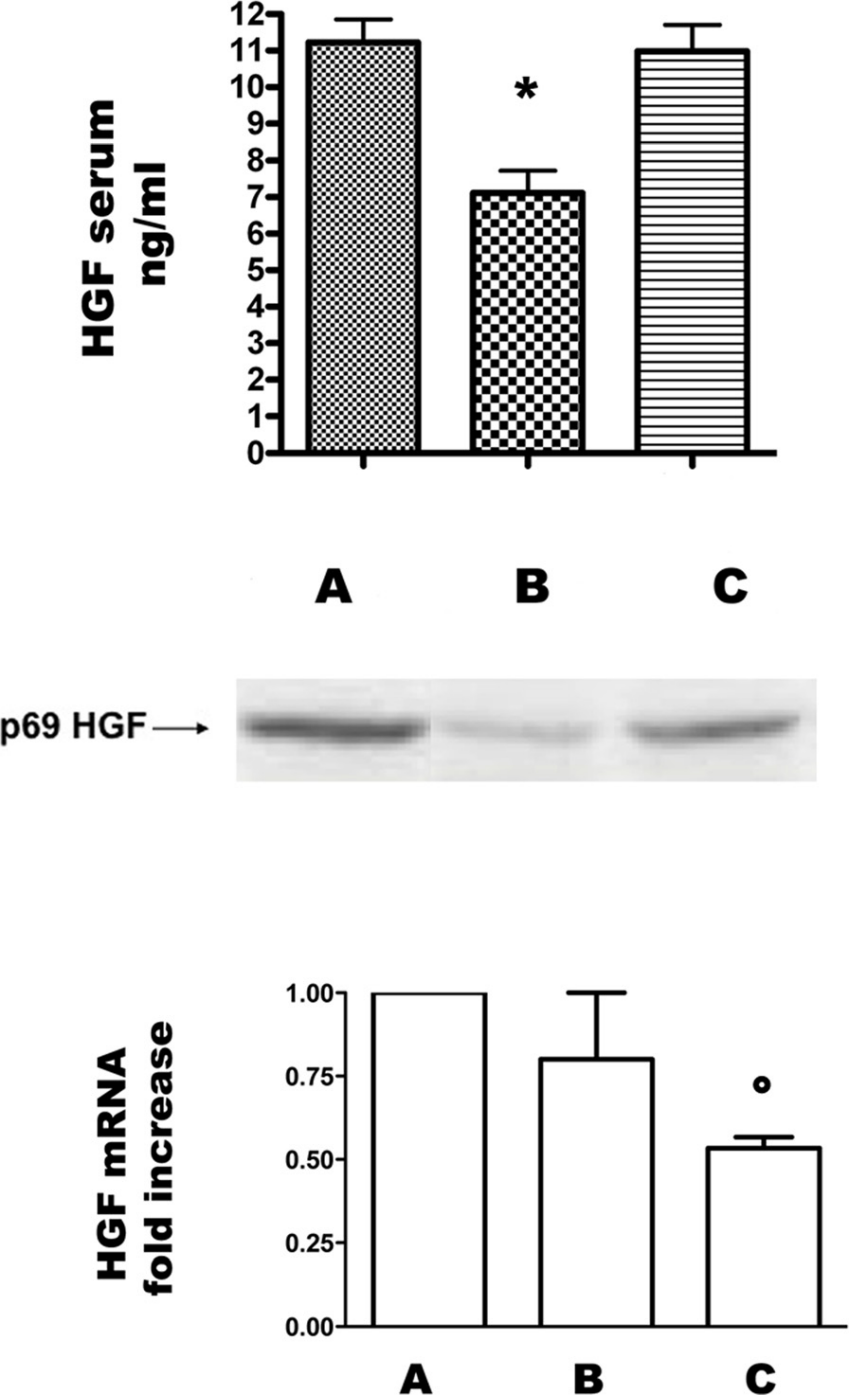
**Serum HGF levels, HGF mRNA and HGF protein in kidney of control and allografted rats on day 7.** Groups are defined as in Figure 8. Upper panel: serum HGF levels in A, B, C groups of rats. Columns are means, Bars are SD. *p < 0.005 vs A and C. Middle panel: Western blot performed with anti-HGF antibody in renal tissue of groups A, B, C. The p69-kDa band represents the α sub of active heterodimer HGF. Lower panel: HGF mRNA expression in renal tissue analyzed by RT PCR in all groups of rats. Columns indicate HGF mRNA expression normalized to the beta-actin expression and converted into fold change. °p < 0.05 vs A.

HGF serum levels significantly decreased in grafted rats that were not injected with MSC compared to control normal rats. MSC treatment returned serum HGF to normal levels (Figure 3, upper panel).

Both HGF protein and HGF mRNA were reduced in grafted kidneys compared to native organs. MSC injection prevented the loss of HGF protein (Figure 3, middle panel), although HGF mRNA levels were lower probably for a feedback mechanism (Figure 3, lower panel).

#### Renal expression of Met

**Figure 4:**
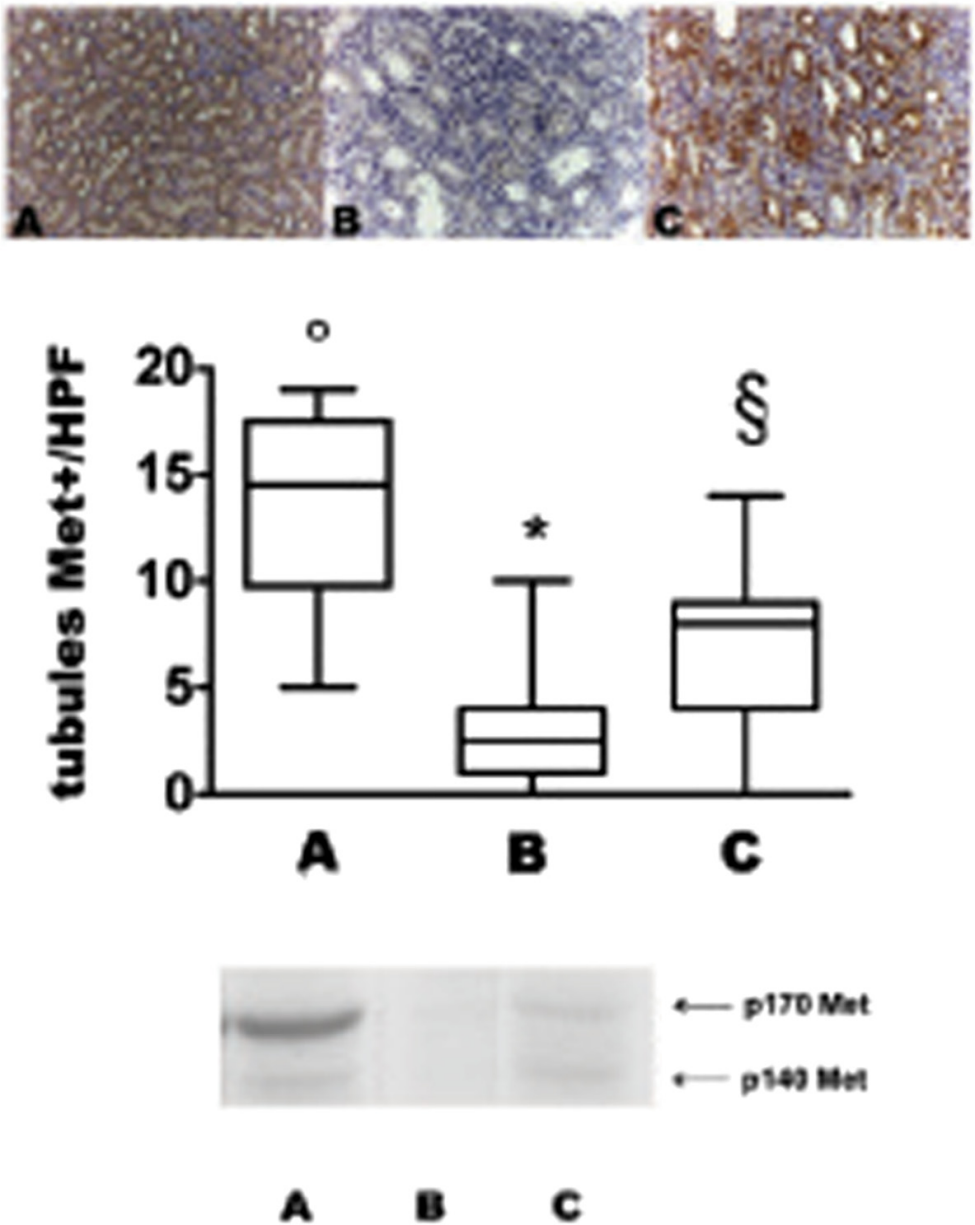
**Met expression in kidney of control and allografted rats on day 7.** Groups are defined as in Figure 8. Upper panel: Met expression in representative renal sections of control and allografted rats on day 7. Panel** A**: normal kidney, Panel** B**: untreated allograft, Panel** C**: MSC treated allograft (Magnification X200). Middle panel: Bars represent the medians of Met positive tubules number/HPF in all groups of rats, boxes represent the 25-75% percentile, whiskers: min to max. °p < 0.001 vs B, ^§^ p < 0.005 vs A, * p < 0.005 vs C. Bottom panel: Western blot performed with anti-Met antibody in renal tissue of all groups of rats. The p170-kDa band represents pro-Met, the p140-kDa band represents mature Met β subunit.

The widespread expression of Met protein in tubules of native kidneys (Figure 4, panel A) was almost completely abrogated in untreated grafted kidneys (Figure 4, panel B). MSC infusion prevented Met loss (Figure 4, panel C). The result was confirmed by Western Blot for Met performed on kidney tissue (Figure 4, lower panel).

### MSP/RON system

#### Renal expression of MSP/RON

**Figure 5:**
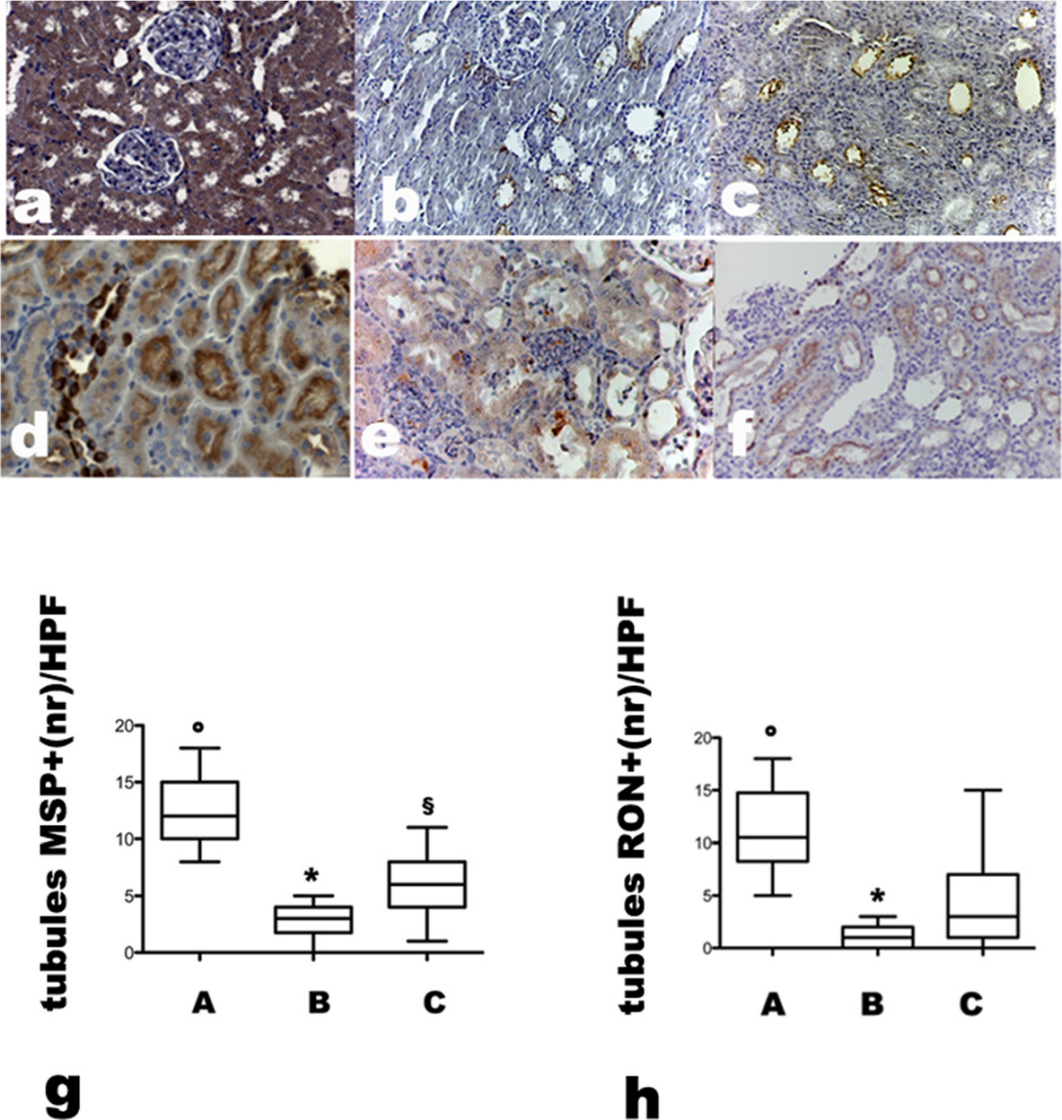
**MSP and RON expression in kidney of control and allografted rats on day 7.** Groups are defined as in Figure 8. Panel** a**: MSP expression in representative renal section of normal kidney, note the strong cytoplasmic pattern of staining, panel** b**: MSP expression in representative renal section of untreated allograft, note the almost complete absence of MSP expression, panel** c**: MSP expression in representative renal section of MSC treated allograft, note the luminal pattern of MSP staining (Magnification X200). Panel** d**: RON expression in representative renal sections of normal kidney, panel** e**: RON expression in representative renal sections of untreated allograft, note the presence of RON positive inflammatory cells and the significant reduction of RON tubular staining, panel** f**: RON expression in representative renal sections of MSC treated allograft, note the reappearance of RON tubular staining and absence of RON positive inflammatory cells (Magnification X200). Panel** g**: Bars represent the medians of MSP positive tubules number/HPF in all groups of rats; Boxes represent 25-75% percentile of MSP positive tubules, whiskers: min to max) i: °p < 0.005 vs B; *p < 0.05 vs C, ^§^ p < 0.01 vs A. Panel** h**: Bars represent the medians of RON positive tubule number/HPF in all groups, boxes represent the 25-75% percentile, whiskers: min to max. *p < 0.01 vs C, °p < 0.005 vs B and C.

Immunohistochemistry showed that MSP was expressed in tubular cells of native kidneys (Figure 5 panel a) and it was undetectable in untreated grafts except for some tubules in which it was distributed with luminal pattern (Figure 5, panel b). MSC restored tubular MSP in transplanted rats, but in a different pattern from normal, i.e. luminal instead of cytoplasmic (Figure 5, panel c). As MSP also its receptor, RON, was diffusely expressed with cytoplasmic pattern in tubules of healthy rats (Figure 5, panel d), while in transplanted rats with acute rejection tubular RON expression was significantly decreased (Figure 5, panel e) and reappeared in tubules of allografts treated with MSC (Figure 5, panel f). In untreated grafts RON was expressed also by some inflammatory cells in the interstitium, but it was absent in infiltrating cells of MSC treated rats (Figure 5, panels e and f).

#### MSP mRNA expression in kidneys

**Figure 6:**
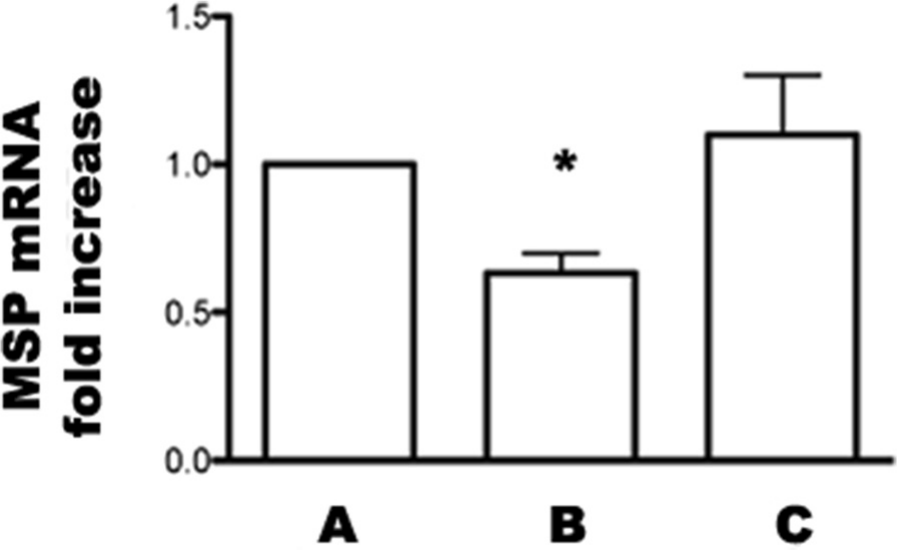
**MSP mRNA expression of control and allografted rats.** Groups are defined as in Figure 8. MSP mRNA expression in renal tissue analyzed by RT PCR in all groups of rats. Columns indicate MSP mRNA expression normalized to the beta-actin expression and converted into fold change. *p < 0.05 vs A and C.

RT-PCR showed a significant decrease of MSP mRNA in the untreated graft. MSC injection was associated with recovery of MSP mRNA expression (Figure 6).

#### MSC express MSP mRNA

We performed in vitro experiments aimed to understand whether MSC are possible producers of MSP.

**Figure 7:**
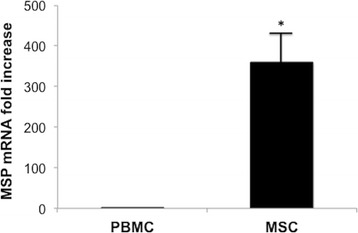
**Semiquantitative MSPmRNA expression in PBMC, MSC in basal condition evaluated by reverse transcription (RT)–PCR that used 1 mg of RNA as the template.** PBMC known for their capacity to produce MSP were used as controls. Columns indicate MSP mRNA expression normalized to the beta-actin expression and converted into fold change. *p < 0.001 vs.

PBMC known to be constitutive MSP producers (44) were used as controls. The expression of MSPmRNA was investigated by quantitative PCR in PBMC and in MSC. The expression of MSP mRNA in PBMC was held as the arbitrary unit. MSC expressed a greater amount of constitutive MSP compared with PBMC (Figure 7).

#### RON mRNA and RON protein expression in monocytes

RON mRNA expression normalized to the beta-actin significantly increased in monocytes stimulated *in vitro* for 6 h with LPS and INF-γ than in monocytes cultured in basal condition (C) respectively (LPS: 3,8 ± 0,5 fold increase, p < 0.001 vs C) (IFN-γ: 70,.03 ± 0,3 fold increase, p < 0.001 vs C ) (data not showed).

RON positive monocytes percentage in basal condition (C) was 28,4 ± 2%, but it increased significantly after *in vitro* monocytes stimulation for 24 h with respectively LPS and INF-γ (LPS: 54,4 ± 4%, p < 0.05 vs C; IFN-ϒ: 56,7 ± 3.5%, p < 0.05 vs C) (data not showed).

## Conclusions

We formerly showed that MSC injected in a rat model of renal transplantation attenuated the severity of rejection and we found also that such effect was independent of MSC endowing in the transplanted kidney. The latter findings indicate that MSC operate through intermediate effectors, as it was elegantly demonstrated by Bi et al. [29] who reproduced MSC effects by substituting MSC for their culture medium.

Aim of the present study was to identify possible effectors of the protection provided by MSC in the renal transplant model. Actually, the study consisted of a straightforward continuation of our former ones in which we demonstrated that MSC prevent the rejection [15,44].

We have pointed out two systems: (i) the cytokine network that regulates the immune response in acute rejection, and (ii) the Scatter Factors systems that have been shown to promote repair and to modulate immune cell traffic in renal tissue in different models of kidney disease [36-42,45]. In addition to confirming that MSC significantly decrease CD4, CD8 cells and monocytes infiltration [15], here we report that on day 7 of grafting in MSC untreated rats, e.g. rats with acute severe rejection IFN-γ and IL-10 levels rose impressively both in serum and in graft tissue, while MSC injection prevented the rise in IFN-γ and simultaneously it caused a further rise in IL-10 levels. Since IFN-γ is a nominal marker of Th1 lymphocyte activity, while IL-10 is Th2 cells product [46,47], these results suggest that MSC reset the balance between the two T helper subpopulations, contrasting the prevalence of Th1 over Th2. MSC effects on T cell subsets included also a rise of Foxp3+ cells number, i.e. Treg lymphocytes in the transplanted kidney. This finding confirms that MSC induce Tregs, as shown in patients with SLE [48] and renal graft [49] in which circulating Tregs rose after MSC infusion and in a mouse model of renal transplantation in which pre-transplant MSC infusion was associated with a significant prolongation of graft survival by Treg-dependent mechanism [16]. Altogether the results of our study suggest that MSC reset T cell subpopulations, decreasing the prevalence of Th1 cells that are main effectors of rejection, increasing the activity of the immunosuppressive Th2 subset and recruiting tolerogenic Tregs. In addition to changing the T cell phenotypic distribution, MSC blocked IL-6 overproduction, a major inflammatory product of monocyte/macrophage cell and effector of acute rejection [50,51]. Therefore, MSC suppressed the cytokines that drive the graft assault by the two major cell effectors of acute rejection, Th1 lymphocytes and monocytes.

A new information given by our study is that MSC have relevant effects on the Scatter Factor systems. In fact, in MSC untreated rats the HGF/Met couple was suppressed in serum (HGF) and in renal tissue (both HGF and Met). MSC prevented such abatement of HGF/Met, thus saving a system that has been proved to protect the kidney in diverse experimental models of renal disease. Actually, HGF has several features that facilitate renal healing: it stimulates proliferation and blocks apoptosis of injured tubular cells, it induces formation of new tubular structures in renal epithelial cells and generates new capillary vessels [52-54], it downregulates the inflammatory and immune response, e.g. by inactivating dendritic cells [55,56], resetting cytokine network and addressing transformation of T cells to the Th2 phenotype [57]. HGF was shown also to interfere with the expression of immune co-stimulatory molecules, and to prolong survival of cardiac allograft by preventing acute rejection in a rodent model [58]. Furthermore, in a model of acute renal rejection, administration of recombinant HGF expanded Treg cell subset [59]. Indeed, there is an impressive overlap between the activities that are attributed to HGF and the changes induced by MSC in grafted rats, e.g. the shift of T cells to a tolerogenic phenotype, the rescue of tubular cells from death and the increase in tubular cell proliferation, that are the most distinctive features of HGF activity on renal tubular cells. Therefore, we believe that HGF/Met is a system that mediates the protective MSC effect on the kidney graft. Interestingly, MSC induced a rise in HGF levels also in another rat model of renal diseases, i.e. anti-Thy 1 nephritis [43] and ischemia-reperfusion injury [60] and in both models HGF overexpression was associated with prevention of renal damage.

In contrast to the abundant literature available on the pleiotropic activities of HGF/Met, the homologous factor MSP and its receptor RON are less known and their role in renal physiology and disease has been scarcely investigated. We have shown that tubular cells produce MSP and that MSP and RON are diffusely expressed in the normal kidney [33]. Studies in vitro have demonstrated that MSP induces in tubular cells proliferation, resistance to apoptosis, migration and branching morphogenesis, i.e. effects that altogether suggest a role of MSP expressed in the kidney as an autocrine/paracrine factor that protects survival and stimulates proliferation of tubular cells. In fact, MSP was shown to attenuate renal injury in the glycerol-induced model of acute renal failure [37]. In the present study we found that MSP and RON were suppressed in renal grafts not injected with MSC i.e. in the setting of unopposed acute rejection. MSC injection had significant effects on MSP/RON system that consisted of *(i)* recovery of MSP mRNA levels in tubular cells, (ii) recovery of MSP and RON expressed in tubular cells with MSP shifted in a different cell location, i.e. on the cell surface rather than inside the cytoplasm, *(iii)* suppression of RON in infiltrating monocyte/macrophage cells. We interpret this combination as a series of actions that benefit the kidney by *(i)* restarting the translation of MSP and the production of MSP protein in an amount that makes it detectable in the tubular cell (ii) shifting the MSP molecule to the cell surface, i.e. in a site where it can meet its receptor and possibly activate it in an autocrine fashion (iii) suppressing RON in monocytes thus restraining their ability to address tubular cells. As for the last effects, in order to understand the underlying mechanism we designed an *in vitro* experiment that demonstrated for the first time that MSC express constitutively mRNA of MSP in amount greater than that expressed by PBMC, i.e. a reference constitutive MSP producer [61]. RON expression in monocytes is induced by IFN-γ. Since MSC suppress IFN-γ both in serum and in renal tissue, it seems reasonable that this mechanism accounts for the absence of RON in monocytes infiltrating the renal graft injected with MSC.

Recent studies in a mouse model of renal transplantation have shown that MSC effects on graft function depend on the time of their injection, i.e. MSC worsen kidney function when injected after grafting, while they prevent rejection and ameliorate renal outcome when administered 1 or 7 days before the grafting [16]. These observations have a relevant impact on deciding the mode of MSC administration that should be used in clinical transplantation. We have chosen to inject MSC into the renal artery soon after reperfusion of the graft for two reasons: (i) to avoid changes in MSC activity caused by their transit in the lung, and (ii) to administer MSC in a setting that is easily reproducible in the circumstances of transplantation from a cadaver donor, in which the identity of the recipient is known just at the last minute. Our demonstration that this mode is feasible offers a potential advantage, mainly because, once our model is proven safe in humans, it allows to deliver MSC to recipients of kidney from cadaver donors that represent the majority of patients crowding the waiting lists. However, the two models are hardly comparable not only because of the different mode of administration, but also because the studies were performed in different species. Nonetheless it is interesting that both of them give useful information to programme use of MSC in humans, and both identify Tregs as effectors of MSC immunosuppressive action. In summary, we have demonstrated that the prevention of rejection provided by MSC is associated with a shift of T cells to an immune suppressive phenotype and with the recruitment of the Scatter Factor systems. These observations identify candidate mediators of MSC activity.

## Methods

### Animals and experimental model

**Figure 8:**
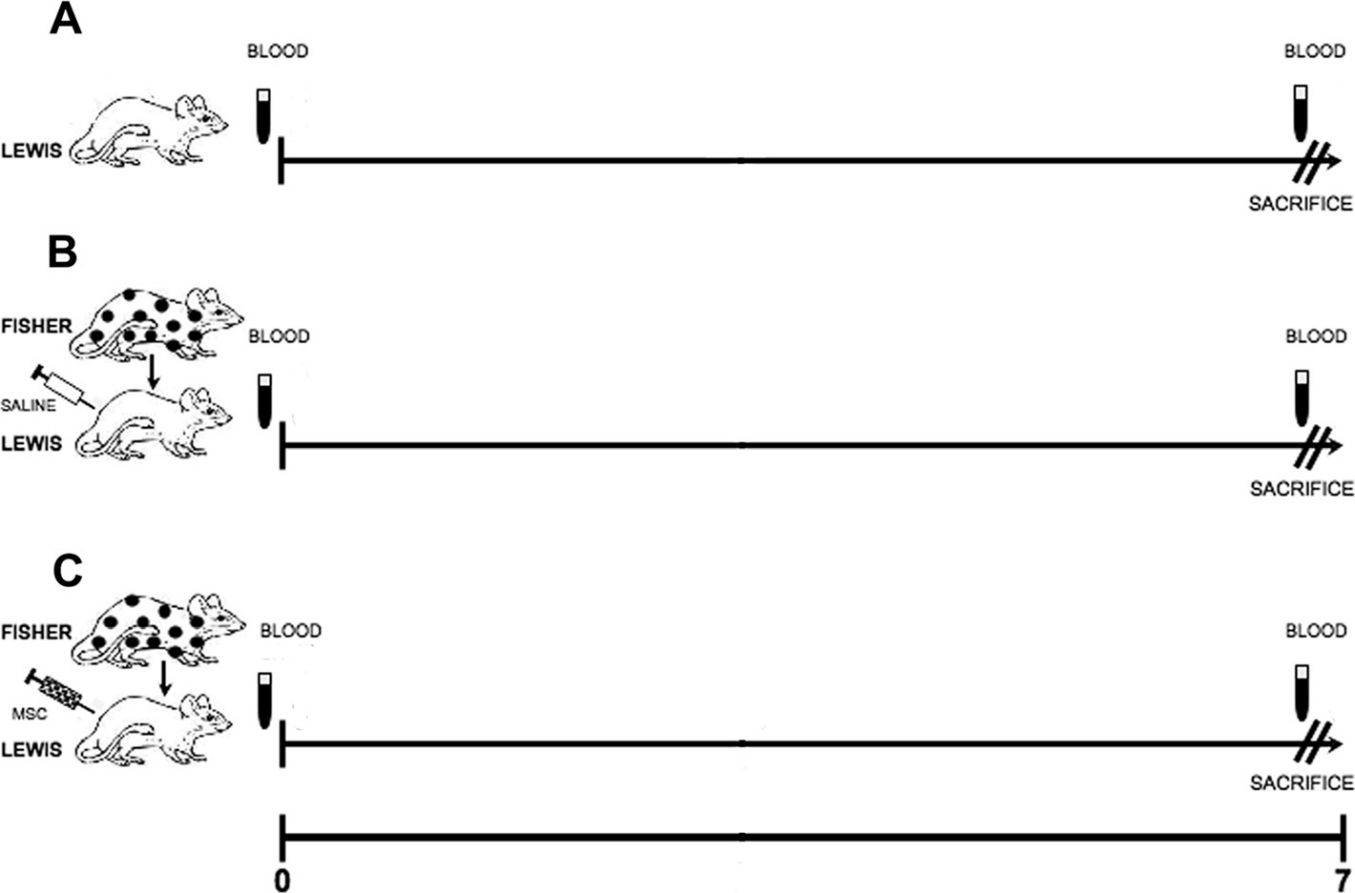
**Experimental design.** We studied a kidney transplant allogeneic model (Fisher to Lewis, 20 rats). 10 allografted rats (group B) were infused into the renal artery soon after graft reperfusion only with saline. 10 allografted rats (group C) were infused into the renal artery with 3 × 10^6^MSC in 1 ml of PBS soon after graft reperfusion. Bilateral nephrectomy was performed in all rats soon before transplantation. 5 healthy rats (Lewis) were the control group (group A). No immunosuppressive therapy was administered. Blood was drawn from the caudal vein at day 0 (the day of transplantation, before the surgery), and at day 7. All the rats were sacrificed at day 7. **A**: healthy rats. **B**: allografted rats treated with saline, **C**: allografted rats treated with MSC.

The present study was carried out as a straightforward continuation of our former studies in a rat model of kidney transplant in which we demonstrated that MSC prevent severe rejection [15,44]. In fact, we used specimens of serum and renal tissue that were sampled from the same rats and were stored on purpose. The design of the animal experiments is summarized in Figure 8. In brief, 11-week old Fisher F344 rats were used as kidney donors, 7-week old Lewis RT1 rats were used as recipients and Transgenic Sprague–Dawley rats (n = 5) expressing Enhanced Green Fluorescent Protein (EGFP) (Japan Slc, Hamamatsu, Japan) were used as MSC donors [62].

We studied an allogeneic model (Fisher F344 to Lewis RT1, 20 rats) of renal transplant in which both native kidneys were excised soon before grafting and no immunosuppressive drug therapy was administered. The experimental group consisted of ten rats that were infused into the artery of the grafted kidney with 3×106 MSC in 1 ml of PBS (Sigma Aldrich, St. Louis, MO, USA) (group C), soon after reperfusion. We used two control groups, one of normal not grafted rats (n = 5, group A), one of grafted rats that were infused into the graft artery with 1 ml of saline (n = 10, group B). Blood was drawn from the caudal vein at days 0 and day 7 and serum was stored at −20°C. All the rats were sacrificed at day 7, the kidneys and spleens were removed and cut in two sagittal halves. One half was fixed in 10% formalin, the other one was frozen in liquid nitrogen.

Animal studies have been conducted after the approval by animal ethical committee of the University of Pavia.

### Cytokine levels

IFN-γ, IL-1, IL-2, IL-4, IL-6, IL-10 serum levels were measured in rats of control group and 7 days after transplantation in allografted rats by ELISA (R&D Systems, Minneapolis, USA). The same cytokines were measured at day 7 in the graft by protein assay (Search Light Rat Cytokine Array), (Pierce Chemical Company, Rockford, IL, USA). All experiments were quadruplicated.

### Renal morphology

#### Tubular necrosis, tubular cell proliferation, MSP, RON, Met, Foxp3, ED1, GFP antigen expression

3-μm thick sections cut along the sagittal plane of formalin-fixed kidney were stained with periodic acid-Schiff, and evaluated by two investigators in double blind fashion, using an Olympus IX8 microscope connected with a CCD camera and software imaging analysis Cell-R (Olympus America, Center Valley, PA, USA).

We scored for primary necrosis, i.e. necrosis that was not associated with tubulitis, 40 nonoverlapping high-power fields in 5 (HPF) not consecutive renal sections for each animal and quantified tubular necrosis as the ratio between necrotic tubules number and total tubules counted. A tubule was counted as having necrosis when tubular cells had one or more of the following features: isometric vacuolization, cell membrane rupture with loss of cytoplasm, nuclear fragmentation, detachment from basal membrane. Tubular cell proliferation was evaluated counting cells expressing Proliferating Cell Nuclear Antigen (PCNA). Renal expression of PCNA, MSP, RON, Met, ED1 antigen, Foxp3 and Green Fluorescent Protein (GFP) were studied by immunohistochemistry in formalin fixed tissue in 10 non-overlapping high-power fields of each section. The sections of paraffin embedded tissue were collected on poly-L-lysine-coated slides (Dako, Carpinteria, CA, USA), they were dewaxed in xylol, passed in a decreasing series of alcohol, and finally rehydrated with distilled water. Endogenous peroxidase was blocked with H_2_O_2_ 3.7% vol/vol followed by H_2_O for 15 min. After 3 washings in 150 mM of PBS the sections underwent microwave antigen retrieval. Subsequently they were exposed overnight at 4°C to the following antibodies: 1) monoclonal mouse anti-PCNA antibody (Ab) (Santa Cruz Biotechnology, Santa Cruz, CA, USA), 1:200; 2) polyclonal goat anti-mouse MSP Ab (Santa Cruz Biotechnology), 1:600; 3) monoclonal mouse anti human RON Ab (Transduction Laboratories, Lexington, KY, USA), 1:750; 4) monoclonal mouse anti-human Met Ab (Novocastra Laboratories Ltd, Newcastle, UK), 1:20, 5) ED1 antigen, 1:80 (Serotec LtD, Oxford, UK), 6) anti Foxp3 Ab, 1:50 (eBioscience Ltd, Hatfield, UK), 7) monoclonal mouse anti-GFP antibody IgG1, diluted 1:1000 (Chemicon International, Temecula, CA, USA). After 3 washings in PBS the immunocomplex was visualized with the biotin-streptavidin-peroxidase complex and 3,3-diaminobenzidine (Dako, Glostrup, Denmark). Sections were faintly counterstained with Harris hematoxylin. Negative controls included both omission of the primary Ab and substitution of IgG for primary antibodies. Positive controls for EGFP positive cells were sections of kidney of EGFP rat. We counted the number of PCNA, Foxp3, GFP positive cells, MSP, RON, Met positive tubules number and RON positive interstitial cells in ten renal sections for each animal.

#### HGF serum levels

HGF serum levels were measured in control and allografted rats 7 days after transplantation by ELISA (Institute of immunology, Tokyo, Japan). All experiments were quadruplicated.

#### Western blot for HGF, Met

Tissue samples were frozen at −70°C in liquid nitrogen immediately following nephrectomy. Tissues were pulverized with a Mikro-Dismembrator (B.Braun Biotech International, Melsungen, Germany) in the presence of liquid nitrogen. The powdered whole tissues were washed twice with PBS and homogenized in ice cold buffer containing 10 mM PIPES, pH 6.8, 100 mmol/L NaCl, 5 mmol/L MgCl_2_, 300 mmol/L sucrose, 5 mmol/L ethylene-glycosbis-(β–aminoethyl ether)-N-N’-tetraacetic acid (DIM buffer), 1% Triton X-100, 100 μmol/L sodium ortho-vanadate, and protease inhibitors (aprotinin 10 μg/mL, pepstatin 10 μg/mL, leupeptin 50 μg/mL, soybean trypsin inhibitor 100 μg/mL phenylmethanesulfonyl fluoride 1 mmol/L) (Sigma-Aldrich). Equal amounts of protein (800 μg), determined using the BCA Protein Assay Reagent Kit (Pierce Chemical Company), were separated on 7,5% sodium dodecylsulfate-polyacrylamide gel electrophoresis and transferred to nitrocellulose Hybond filters (Amersham - GE Healthcare, Little Chalfont, Buckinghamshire, UK). Filters were probed with goat polyclonal anti HGF α Ab (c-20, Santa Cruz) that recognizes the α subunit of active heterodimer HGF, the rabbit polyclonal anti Met Ab that recognizes pro-Met and mature Met β subunit (c-28, Santa Cruz). The specific binding was detected by the enhanced chemiluminescence system ECL-Plus (Pierce Chemical Company).

### In vitro experiments

#### Cell cultures

Peripheral blood mononuclear cells (PBMC) were isolated from rat blood by standard Ficoll–Hystopaque density gradient separation (Sigma-Aldrich). Monocytes were collected after 45 min of adherence to plastic culture plates and resuspended in RPMI 1640 medium, FCS 10%, and penicillin/streptomycin 1% (Invitrogen, Carlsbad, CA, USA) at 37°C in humidified 5% CO2 atmosphere.

To understand whether IFN-γ modified monocytes RON expression, we studied RON mRNA and RON protein in circulating monocytes soon after sampling and in monocytes cultured in absence and presence of LPS (10 μg/ml, Sigma - Aldrich) and INF-γ (10 ng/ml, R&D Systems) for 6 h and for 24 h respectively.

#### Monocytes RON mRNA, mesenchymal stem cells MSP mRNA, Renal MSP mRNA and HGF mRNA expression

Total RNA was extracted from renal tissue and cell cultures using respectively trizol method and guanidine-based RNeasy® Mini Kit (QIAGEN GmbH, Hilden, Germany). All RNA was treated with DNase from RNase-Free DNase Set (QIAGEN) and dissolved in nuclease free water. Extracted RNA was tested for quantity and integrity by spectrophotometric analysis (NanoDrop - Thermo Scientific, Waltham, MA, USA). A total of 1 μg of RNA per condition was reverse transcribed into complementary DNA (cDNA) through 1st Strand cDNA Synthesis Kit for Real Time (RT) -PCR (AMV) (Roche Applied Science, Penzber, Germany). cDNA was used to perform RT-PCR analysis in 96-well optical reaction plates, using ABI prism 5700 (Applied Biosystems, Foster City, CA, USA) and the 5-exonuclease assay (TaqMan technology) in a volume of 25 μl reaction containing TaqMan Universal Master Mix, optimized concentrations of FAM-labelled probe, and specific forward and reverse primers for beta-actin, HGF, MSP and RON selected from Assay on Demand (Applied Biosystems). Controls included RT-PCR with water replacing cDNA. The results were analysed using a comparative method, and the values were normalized to the beta-actin expression and converted into fold change, as previously described [63].

#### Expression of RON receptor in monocytes

A total of 10 ml of EDTA – anticoagulated peripheral blood and 3X10^5^ monocytes were incubated for 30 min with 10 μl of mouse anti rat RON mAb (Transduction Laboratories) diluted 1:10. Subsequently the samples were incubated for 30 min with 2 μl of goat anti mouse PE conjugated followed by incubation for 30 min with 2 μl of mouse anti rat CD11b FITC conjugated (BD Biosciences, San Josè, CA) to identify rat monocytes. Before examination samples of whole blood were treated with lysis buffer. All samples were resuspended in 500 μl of PBS and analyzed in FACScan operating with Cell Quest 3.3 software (BD Biosciences). Goat PE- conjugated IgG antibodies were used as negative controls.

#### Statistical analysis

ANOVA followed by the Newman-Keuls test or Kruskal-Wallis test and Student t test were used for comparison of the medians and means. All data was analysed using Graph Pad Prism software.

## Abbreviations

Ab: Antibody
ANOVA: Analysis of variance
BCA: Bicinchoninic acid
CD: Cluster differentiation
cDNA: Complementary DNA
EDTA: Ethylenediamine tetraacetic Acid
EGFP: Enhanced GFP
ELISA: Enzyme-linked immunosorbent assay
FAM: 6 carboxyfluorescein
FCS: Fetal calf serum
FITC: Fluorescein isothiocyanate
Foxp3: Forkhead box p 3
g: Gram(s)
GFP: Green fluorescent protein
HGF: Hepatocyte growth factor
HPF: High power field
IFN- γ: Interferon gamma
LPS: Lipopolysaccharide
M: Molar
mmol: Millimole(s)
MSC: Mesenchymal stromal cells
MSP: Macrophage stimulating protein
nr: Number
PBMC: Peripheral blood mononuclear cell(s)
PBS: Phosphate buffered saline
PCNA: Proliferating cell nuclear antigen
PE: Phycoerythrin
PIPES: Piperazine N,N’ bis(2 ethanesulfonic acid)
RT-PCR: Reverse transcriptase polymerase chain reaction
SD: Standard deviation
SF: Scatter factor
SLE: Systemic lupus erythematosus
Th: T helper
Treg: Regulatory T cell
Vol: Volume
α: alfa
β: beta
μg: Microgram(s)
μmol: Micromole(s)
